# Clinical evaluation of Ayush-SS granules in exclusively breastfeeding mothers with insufficient lactation: A randomized, double-blind, placebo-controlled trial

**DOI:** 10.1186/s13006-025-00721-9

**Published:** 2025-04-05

**Authors:** Upma Saxena, Sarada Ota, Shivshankar Rajput, Bhavna Anand, Arunabh Tripathi, Richa Singhal, Adarsh Kumar, Abhishek Lachyan

**Affiliations:** 1 Department of Obstetrics & Gynecology, VMMC and Safdarjung Hospital, New Delhi, India; 2 Central Ayurveda Research Institute, Bhubaneswar, India; 3 Central Ayurveda Research Institute, Punjabi Bag, New Delhi, India; 4 Department of Pediatrics, VMMC and Safdarjung Hospital, New Delhi, India; 5 Central Council for Research in Ayurvedic Sciences, New Delhi, India

**Keywords:** Ayush-SS granules, Breastfeeding, Complementary medicine, Neonatal nursing

## Abstract

**Background:**

Breast milk is essential for infant growth and development, with exclusive breastfeeding (EBF) recommended for the first six months. Many mothers experience insufficient milk production, leading to early supplementation or cessation of breastfeeding. Ayurvedic formulations, such as Ayush-SS granules, have been proposed to enhance lactation. This study evaluated Ayush-SS granules as a galactagogue, with EBF at six months as the primary outcome. Secondary outcomes included infant weight gain over 90 days and maternal perceptions of lactation sufficiency.

**Methods:**

A double-blind, randomized, placebo-controlled trial was conducted between 1 February 2021 and 31 January 2023 at Vardhman Mahavir Medical College and Safdarjung Hospital, Delhi, India. A total of 210 lactating mothers (aged 20–40 years) with full-term singleton infants (≥ 37 weeks, ≥ 2.5 kg) experiencing insufficient lactation were enrolled. Infants who had not regained birth weight by day 14 or gained < 20 g/day after day 15 were included. Participants were randomized (1:1) to receive 6 g of Ayush-SS granules or placebo twice daily for 12 weeks. All participants received standardized breastfeeding counseling. Safety monitoring was conducted, and an intention-to-treat analysis was performed.

**Results:**

At six months, 74 of 106 (69.8%) infants in the Ayush-SS group were exclusively breastfed, compared to 47 of 105 (44.8%) in the placebo group (*p* < 0.001). After 90 days, 81 of 106 infants (76.5%) in the Ayush-SS group gained ≥ 20 g/day compared to 44 of 105 (41.8%) in the placebo group (*p* < 0.001). Maternal perceptions of insufficient lactation were significantly lower in the Ayush-SS group (15.3% vs. 58.2%). No severe adverse events related to the intervention were observed.

**Conclusion:**

Ayush-SS granules significantly improved EBF rates at six months, maternal perceptions of lactation sufficiency, and infant weight gain. These findings suggest their potential as a galactagogue for mothers with insufficient milk production. Further research is needed to explore long-term effects and maternal dietary influences.

**Clinical trial registration:**

CTRI/2019/08/020579 [Registered on: 06/08/2019].

## Background

Milk insufficiency, defined as the inability to produce enough breast milk to meet an infant’s nutritional needs for exclusive breastfeeding, is reported to affect 60–90% of mothers in low- and middle-income countries [1]. However, this broad range reflects regional variations and differing assessment methods. Many mothers perceive their milk supply to be inadequate even when it is sufficient, contributing to overestimated rates.

In this study, documented insufficient lactation was a key inclusion criterion, requiring clinical confirmation rather than reliance on maternal perception. This stricter definition reduced the number of eligible participants compared to broader prevalence estimates. While breastfeeding counseling remains the gold standard for addressing milk insufficiency, particularly in the absence of underlying health conditions, its implementation is challenging in low-resource settings. Many mothers cite inadequate milk production as a primary reason for early formula introduction or breastfeeding cessation [2].

Ayurvedic texts extensively discuss lactation, including breast milk formation (stanya), milk ejection (stanyapravriti), and the use of galactagogues (stanyaviridhi dravyas) to enhance milk production [3, 4]. Specific herbs are believed to regulate lactation-related hormones and improve maternal digestion, which can indirectly support breast milk production. Ayush-SS Granules, formulated based on Ayurvedic principles, are traditionally used to promote lactation in mothers experiencing insufficiency.

### Granule composition and dosage

Ayush-SS Granules were formulated as a herbal supplement incorporating traditionally used Ayurvedic ingredients (Table 1). Each 6 g dose contained Shatavari, an adaptogenic herb with estrogenic properties, hypothesized to regulate lactation-related hormones [5]. It also included Shatpushpa and Shweta Jeerak, which support digestion and nutrient absorption, both of which are critical for optimal milk production [6, 7]. Additionally, Sukshma Ela was incorporated due to its traditional use in enhancing digestive health and reducing inflammation.

**Table 1:** Granule composition and dosage

Ingredient	Botanical Name	Amount per 6 g Dose
Shatavari	*Asparagus racemosus Willd.*	2 g
Shatpushpa	*Anethum graveolens Roxb. ex Flem.*	1.5 g
Shweta Jeerak	*Cuminum cyminum L.*	1 g
Sukshma Ela	*Elettaria cardamomum Maton*	0.5 g
Other Excipients (e.g., sugar)		1 g

Participants in the intervention group received 6 g of Ayush-SS Granules twice daily (6 g BD) with warm milk for 12 weeks. The placebo group received a visually identical, pharmacologically inert powder, composed of I.P. grade starch and minimal sucrose, ensuring it contained no active herbal components [7].

The placebo was designed to closely mimic the intervention granules in appearance, texture, and mouthfeel while remaining biologically inactive. Given that the active granules contained starch-based excipients along with the herbal ingredients, their natural taste profile was mild. To maintain blinding, the placebo included a small amount of sucrose, ensuring a neutral but comparable taste. Both formulations were mixed with milk before consumption to standardize the sensory experience [7]. The aim of this study is to evaluate the galactagogue effect and clinical safety of Ayush-SS granules in lactating mothers with insufficient lactation.

## Methods

### Trial design and site

This double-blind, randomized controlled trial was conducted between 1 February 2021 and 31 January 2023 at Vardhman Mahavir Medical College and Safdarjung Hospital, Delhi, India.

### Study population and sample size

The trial targeted lactating mothers experiencing insufficient breast milk production. It was hypothesized that Ayush-SS Granules would increase the proportion of mothers exclusively breastfeeding at six months postpartum from 55 to 75%, with 80% power and 5% Type I error rate. The calculated sample size was 85 participants per group. To account for 20% attrition, the final enrollment target was 105 per arm, totaling 210 participants.

### Definition of insufficient lactation

Self-reported milk insufficiency was confirmed using objective clinical criteria. Infants were classified as experiencing insufficient lactation if they failed to regain their birth weight by 14 days postpartum, exhibited a weight gain of less than 20 g/day between days 15 and 90 of life, or had fewer than six urinations per day.

### Eligibility criteria

#### Inclusion criteria

Participants were lactating mothers aged 20–40 years, either primiparous or multiparous, with full-term singleton infants (≥ 37 weeks gestation, birth weight ≥ 2.5 kg) and clinically confirmed insufficient lactation within the past three months. Written informed consent was obtained.

#### Exclusion criteria

Mothers were excluded if they had medical conditions affecting lactation (e.g., active herpes, breast abscess, retracted nipples, chronic illnesses) or were on medications influencing milk production. Infants with conditions impairing breastfeeding were also excluded.

### Randomization and blinding

Participants were randomized in a 1:1 ratio to receive Ayush-SS Granules or placebo, using computer-generated randomization. Medication boxes were identical in appearance, ensuring blinding for both participants and investigators.

### Study interventions

Participants in the intervention group received 6 g of Ayush-SS Granules, equivalent to six capsules per dose, administered twice daily with milk for 12 weeks. The control group received an identical placebo composed of I.P. grade starch, ensuring the absence of active herbal ingredients. Both groups underwent standardized breastfeeding counseling, following the Academy of Breastfeeding Medicine’s Clinical Protocol #3: Supplementary Feedings in the Healthy Term Breastfed Neonate [8] which provides evidence-based recommendations to support optimal lactation practices.

### Laboratory investigations

Maternal assessments were conducted at baseline and after 12 weeks, including a complete blood count (CBC), erythrocyte sedimentation rate (ESR), thyroid function tests, fasting blood sugar, liver function tests, and kidney function tests. However, prolactin levels were not measured due to logistical constraints.

### Breastfeeding measurement definitions

Exclusive breastfeeding was defined as infants receiving only human milk in the previous 24 h, with no supplementation of formula, water, or solid foods, except for prescribed vitamins or medications. Bottle feeding referred to infants receiving expressed breast milk without the addition of formula, water, or solid foods.

### Outcome measures

The primary outcome was the percentage of mothers exclusively breastfeeding at six months postpartum.

### Sociodemographic and study variables

Maternal variables included age, education, and dietary habits, specifically assessing vegetarian or non-vegetarian diet patterns, frequency of dairy consumption, and intake of galactagogue-rich foods. Infant variables encompassed age, birth weight, gestational age, and growth parameters. Breastfeeding-related variables assessed exclusivity, duration, and the presence of milk insufficiency.

### Data collection and surveys

Data were collected from 1 March 2021 to 13 October 2022 through structured surveys and clinical assessments. Follow-up visits were conducted at 1, 3, and 6 months postpartum, during which infant weight and urination frequency were recorded. Survey instruments were piloted and validated, with modifications made for clarity.

### Research arms

Group I (Ayush-SS Granules) received 6 g twice daily with milk, while Group II (Placebo) received an identical formulation in appearance and taste but without active ingredients.

### Safety monitoring

Adverse events were systematically recorded throughout the trial. Participants reported any side effects, and research assistants conducted follow-up assessments to identify new health concerns. A comparative safety analysis between groups was performed using chi-square or Fisher’s exact tests, with adverse events classified by severity. An Independent Data Monitoring Committee reviewed safety data every 6 months to ensure participant well-being.

### Data collection and management

Follow-up visits were scheduled at 2-week intervals, typically at 1, 3, and 6 months postpartum. Adherence was monitored, and deviations were recorded.

### Steps to minimize dropout

To enhance participant retention, reminder phone calls were made before scheduled visits to ensure adherence. Participants received INR 300 in cash along with milk packets to support maternal nutrition as an incentive. Additionally, a dedicated research team member provided assistance with logistical concerns, ensuring continuous engagement throughout the study.

### Handling dropout data

Data were analyzed using an as-treated approach, excluding 46 mother-baby pairs who did not complete the study. To assess potential biases, dropout characteristics were compared between groups. Sensitivity analyses were performed to evaluate the robustness of the findings and ensure the validity of the results.

### Baseline data collection

Comprehensive clinical history, sociodemographic data, and feeding practices were documented at enrollment. Physical examinations included maternal vital signs and infant growth parameters (weight, length, and head circumference) assessed using WHO growth charts. Developmental milestones, urination frequency, sleep adequacy, and stool consistency were evaluated at each follow-up visit. Drug compliance was closely monitored, with all data systematically recorded in case report forms (CRFs).

### Ethics approval and trial registration

The study protocol was approved by the Institutional Ethics Committee of Vardhman Mahavir Medical College and Safdarjung Hospital, New Delhi (Protocol V.5.0, dated 19.06.2019; Approval no. IEC/VMCC/SIH/Project/2019-05/26).

The study was prospectively registered with the Clinical Trials Registry of India (CTRI/2019/08/020579 on 06/08/2019).

### Statistical analysis

Data were entered into MS Excel, validated, and analyzed using SPSS (version 29.0). Categorical variables were presented as frequencies (%) and compared using chi-square tests. Binary outcomes at multiple time points were analyzed using Cochran’s Q test to assess changes over time.

## Results

**Fig. 1:**
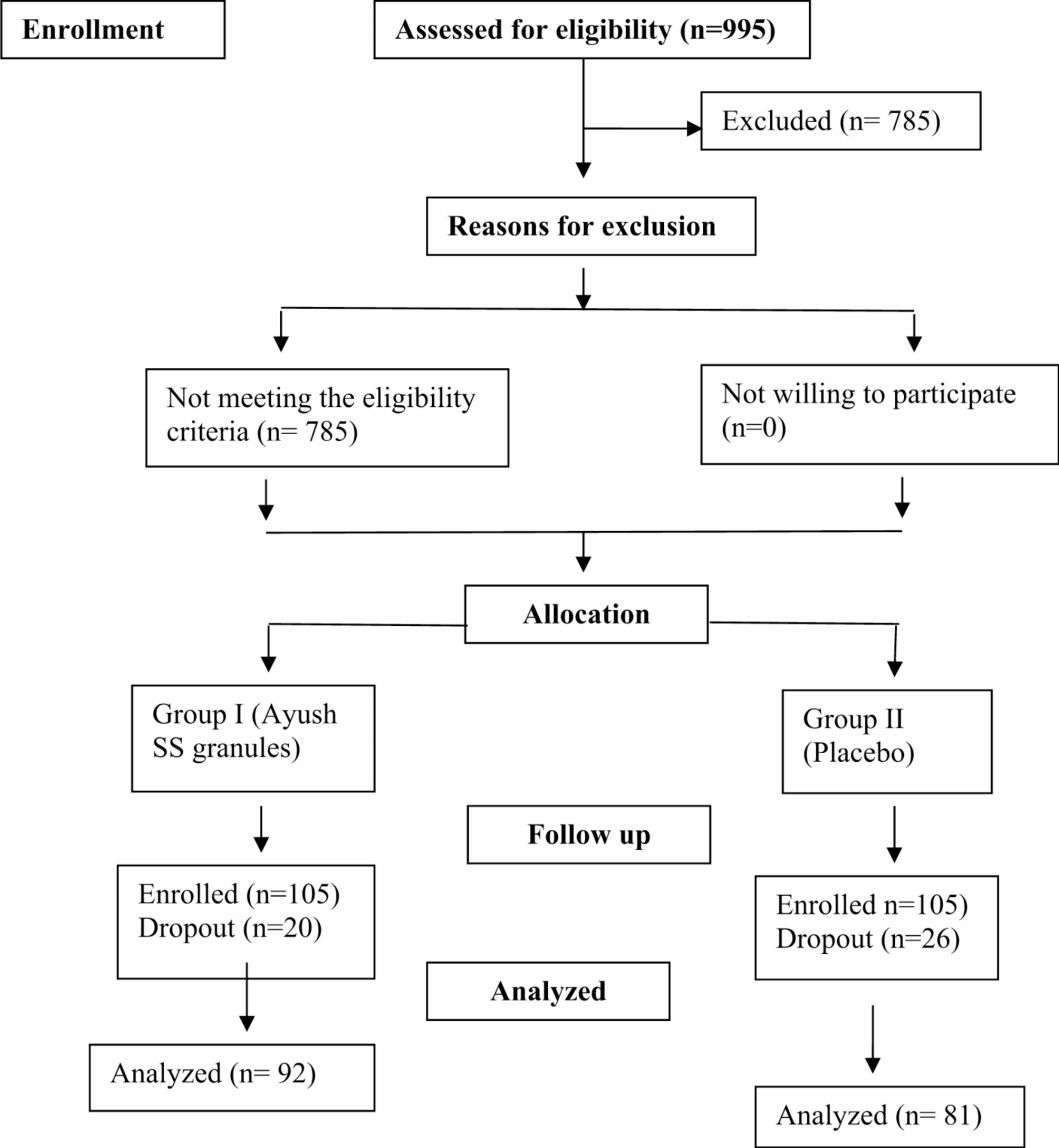
Flow of participants through the study

A total of 210 participants were recruited for the study, achieving the target sample size. The flow of participants is depicted in Fig. 1. Data analysis included 92 participants in Group I (Ayush-SS Granules) and 81 participants in Group II (Placebo). While 20 participants in Group I and 26 in Group II dropped out, data from 13 participants in Group I and 24 in Group II were excluded, while data from 7 to 2 participants were imputed in Groups I and II, respectively.

### Baseline characteristics

Table 2 presents the baseline characteristics of participants in both groups. A total of 210 participants were recruited, and 173 were included in the final analysis (92 in the Ayush-SS Granules group and 81 in the Placebo group). The two groups were comparable in terms of maternal age, education level, and dietary habits. Infant characteristics, including gestational age at birth and birth weight, were also similar.

**Table 2:** Baseline comparison of the participants

Baseline Characteristics	Group I (Ayush SS Granules) (*n* = 105)	Group II (Placebo) (*n* = 105)	Between-group *p*-value#
**Age of Mother (in years)**	25.85 ± 3.880	26.27 ± 4.366	0.463^$^
**Age of Infant (in months)**			
Newborns (< 1 month)	4 (3.8%)	8 (7.6%)	0.234
Infants (1–6 months)	101 (96.2%%)	97 (92.4%)	
**Weight of mother**	57.14 ± 9.006	57.29 ± 9.762	0.904^$^
**Educational Status of Mother**			
Illiterate	3 (2.9%)	2 (1.9%)	0.506^#^
Read and write	22 (21.0%)	21 (20.0%)	
Primary School	29 (27.6%)	30 (28.6%)	
High School	36 (34.3%)	28 (26.7%)	
Graduate and above	15 (14.3%)	24 (22.9%)	
**Diet**			
Non-vegetarian	40 (38.1%)	39 (37.1%)	0.887^#^
Vegetarian	65 (61.9%)	66 (62.9%)	
**Gestational Age at Birth (37–41 weeks)**	38.71 ± 0.917	38.90 ± 1.471	0.261^$^
**Weight (in Kg) of the Baby at Baseline**	4.13 ± 0.879	4.07 ± 0.808	0.640^$^

Values are given as n (%) or mean ± SD

# Compared using chi-square test/ Fisher exact test

$ Compared using independent sample t-test

At enrollment, 35% of participants were exclusively breastfeeding, while 65% were primarily breastfeeding with occasional supplementation.

### Primary and secondary outcomes

Table 3 presents the effects of the intervention on key outcome measures over time.

**Table 3:** Comparison of the effect of treatment on outcome parameters in both groups

Parameter	Baseline	15th Day	30th Day	45th Day	60th Day	75th Day	90th Day
**Proportion of mothers reporting insufficient lactation (%)**							
**Group I**	92 (100.0%)	25 (27.2%)†	21 (22.8%)†	19 (20.7%)†	16 (17.4%)†	14 (15.2%)†	14 (15.2%)†
**Group II**	81 (100.0%)	63 (77.8%)	52 (64.2%)	51 (63.0%)	48 (59.3%)	46 (57.5%)	46 (56.8%)
***p***-**value**	—	< 0.001†	< 0.001†	< 0.001†	< 0.001†	< 0.001†	< 0.001†
**Proportion of babies gaining ≥ 20 g/day (%)**							
**Group I**	—	69 (75.0%)†	69 (75.0%)†	72 (78.3%)†	74 (80.4%)†	74 (80.4%)†	70 (76.1%)†
**Group II**	—	34 (42.0%)	33 (40.7%)	27 (33.3%)	28 (34.6%)	29 (35.8%)	35 (43.2%)
***p***-**value**	—	< 0.001†	< 0.001†	< 0.001†	< 0.001†	< 0.001†	< 0.001†
**Infant weight (kg)**							
**Group I**	4.07 ± 0.83	4.49 ± 0.83†	4.89 ± 0.79†	5.30 ± 0.76†	5.71 ± 0.73†	6.12 ± 0.73†	6.53 ± 0.77†
**Group II**	4.07 ± 0.77	4.33 ± 0.89	4.70 ± 0.79	4.99 ± 0.81	5.27 ± 0.82	5.46 ± 0.98	5.86 ± 0.85
***p***-**value**	—	< 0.001†	< 0.001†	< 0.001†	< 0.001†	< 0.001†	< 0.001†

Notes:

• (†) Statistically significant differences at *p* < 0.05

• Between-group comparisons were performed using the Generalized Estimating Equation (GEE) model

The proportion of mothers reporting insufficient lactation significantly decreased in both groups (*p* < 0.001). However, Group I exhibited a more pronounced reduction, dropping from 100% at baseline to 15.2% at 90 days, compared to 56.8% in Group II.

Weight gain of more than 20 g/day was consistently higher in Group I, peaking at 80.4% by the 60th and 75th days, whereas Group II remained below 44% throughout (*p* < 0.001). Infant weight gain was significantly greater in Group I at all time points (*p* < 0.001), with mean infant weight reaching 6.53 ± 0.774 kg in Group I and 5.86 ± 0.851 kg in Group II by day 90.

At six months postpartum, 84.8% of mothers in the Ayush-SS group were exclusively breastfeeding, compared to 43.2% in the placebo group.

### Infant behavior and activity

Table 4 compares infant behavior and activity patterns between groups.

**Table 4:** Comparison of other measures of babies in the study groups

Parameters		Baseline	15th day	30th day	45th day	60th day	75th day	90th day	*p*-value
**No. of hours baby slept per day**	**Group I**	11.08 ± 2.392	10.38 ± 2.431	9.83 ± 2.366	9.58 ± 2.269	9.40 ± 2.244	9.30 ± 2.208	9.23 ± 2.239	< 0.001 (*)
	**Group II**	10.67 ± 2.324	9.59 ± 2.279	9.04 ± 1.965	8.81 ± 1.810	8.68 ± 1.731	8.48 ± 1.817	8.16 ± 1.647	< 0.001 (*)
	***p***-**value**	0.082							
**No. of times baby urinated per day**	**Group I**	6.67 ± 1.597	7.71 ± 1.134	7.88 ± 1.397	7.92 ± 1.416	7.88 ± 1.656	7.86 ± 1.608	7.92 ± 1.666	< 0.001(*)
	**Group II**	6.63 ± 1.479	6.48 ± 1.314	6.57 ± 1.341	6.52 ± 1.370	6.42 ± 1.439	6.43 ± 1.508	6.40 ± 1.366	0.546
	***p***-**value**	0.003 (*)							
**Proportion of babies crying even after breast feeding** ^**$**^	**Group I**	89 (96.7%)	23 (25.0%)	19 (20.7%)	20 (21.7%)	17 (18.5%)	17 (18.5%)	17 (18.5%)	< 0.001 (*)
	**Group II**	77 (96.3%)	57 (70.4%)	51 (63.0%)	51 (63.0%)	50 (61.7%)	50 (61.7%)	50 (61.7%)	< 0.001 (*)
	***p***-**value**	< 0.001 (*)							

$ Within group *p*-value compared using Cochran - Q test, while between group comparison was done using GEE

# Within and between group comparison done using Repeated Measure ANOVA, (*) denotes significant *p*-value at α = 0.05

Sleep duration per day declined significantly over time in both groups (*p* < 0.001). Although Group I babies consistently slept longer than those in Group II, the difference was not statistically significant (*p* = 0.082).

Urination frequency increased significantly in Group I (*p* < 0.001), whereas no significant change was noted in Group II (*p* = 0.546). Group I babies urinated more frequently than those in Group II, with a statistically significant difference (*p* = 0.003).

The proportion of infants crying after breastfeeding decreased significantly in both groups (*p* < 0.001). However, the reduction was more pronounced in Group I (18.5% by day 90) compared to Group II (61.7%), with the between-group difference being highly significant (*p* < 0.001).

### Safety profile

Table 5 presents safety assessments based on maternal laboratory parameters at baseline and day 90.

**Table 5:** Safety profile of the participants (Mothers) in both the study groups

Lab parameters		Group I	Group II
**Haemoglobin (g/dl)**	Baseline	11.78 ± 1.536	11.61 ± 1.363
	90th day	11.88 ± 1.226	11.99 ± 1.303
	*p*-value^#^	0.602	0.040 (*)
**Liver Function Test (LFT)**			
**Total billirubin (mg/dl)**	Baseline	0.59 ± 0.284	0.66 ± 0.341
	90th day	0.63 ± 0.345	0.61 ± 0.301
	*p*-value^#^	0.575	0.287
**AST (IU/L)**	Baseline	41.84 ± 20.876	36.12 ± 15.277
	90th day	39.00 ± 20.880	31.69 ± 15.261
	*p*-value^#^	0.317	0.035 (*)
**ALT (IU/L)**	Baseline	52.11 ± 31.734	40.84 ± 18.578
	90th day	47.40 ± 37.812	38.80 ± 20.822
	*p*-value^#^	0.289	0.398
**S.Alkaline Phosphatase (U/L)**	Baseline	131.77 ± 55.980	123.95 ± 59.998
	90th day	118.07 ± 42.907	118.80 ± 51.852
	*p*-value^#^	0.009 (*)	0.240
**Renal Function Test (RFT)**			
**Blood Urea (mg/dl)**	Baseline	24.38 ± 8.519	23.25 ± 6.314
	90th day	24.59 ± 7.235	23.24 ± 6.736
	*p*-value^#^	0.745	0.988
**S.Creatinine (mg/dl)**	Baseline	0.75 ± 0.257	0.72 ± 0.167
	90th day	0.77 ± 0.184	0.77 ± 0.200
	*p*-value^#^	0.579	0.030 (*)

# Within group *p*-value compared using paired sample t-test

(*) denotes significant *p*-value at α = 0.05

Significant improvements were observed in hemoglobin levels (*p* = 0.040) and AST reduction (*p* = 0.035) in Group II, whereas Group I showed a significant decrease in S. Alkaline Phosphatase (*p* = 0.009). Serum creatinine levels slightly but significantly increased in Group II (*p* = 0.030), though no other renal or hepatic function parameters showed notable changes in either group.

## Discussion

The findings from this randomized, double-blind, placebo-controlled trial indicate that Ayush-SS granules significantly enhance breast milk production, infant weight gain, and are well tolerated by mothers with documented insufficient lactation.

### Efficacy of Ayush-SS granules

The administration of Ayush-SS granules led to a noticeable increase in milk secretion as early as 15 days after the initiation of the intervention. This early response is particularly important, as the first six months postpartum are crucial for ensuring adequate nutrition to support optimal infant growth. The greater weight gain observed in infants in the intervention group compared to the placebo group suggests that the granules effectively increased milk volume. However, further research is needed to determine whether they had any impact on the nutritional composition of breast milk.

Additionally, infants in the Ayush-SS group exhibited fewer crying episodes after breastfeeding, indicating greater satiety and reduced hunger cues. These infants also showed longer sleep durations, which is recognized as an indicator of adequate nutrition and overall well-being. Improved sleep duration is associated with better growth and developmental outcomes in infants, further reinforcing the potential benefits of Ayush-SS granules in supporting lactation.

### Safety and tolerability

Throughout the trial, all maternal laboratory parameters remained within the normal range in both groups by the study’s conclusion, confirming the safety of Ayush-SS granules for lactating mothers. Although adverse events (AEs) were reported in both groups, they were mild and resolved without requiring medical intervention. The most common AE in the Ayush-SS group was vomiting, reported by one participant, whereas in the placebo group, two participants experienced vomiting and skin rashes. No serious adverse events occurred, reinforcing the tolerability of the intervention.

### Comparison with existing literature

These findings align with previous research on galactagogue herbal formulations, which have demonstrated efficacy in promoting lactation through hormonal regulation and improved digestion. The key ingredients in Ayush-SS granules, such as Shatavari (Asparagus racemosus) and Shatpushpa (Anethum graveolens), have been traditionally used to stimulate prolactin secretion and support breast tissue development. Additionally, their digestive and carminative properties may enhance nutrient absorption, further contributing to improved lactation outcomes.

Previous studies have reported that Shatavari acts as a phytoestrogen, supporting milk production by modulating prolactin levels, while Shatpushpa has been associated with increased milk yield and improved maternal nutritional status. These findings are consistent with our study results, which demonstrated a significant improvement in lactation parameters in the intervention group compared to placebo. However, further clinical trials with larger sample sizes and biochemical assessments of breast milk composition are needed to validate these findings [9–12].

### Implications for clinical practice

The results of this study underscore the potential of Ayurvedic interventions such as Ayush-SS granules in addressing documented insufficient milk production in a safe and effective manner. Given their observed benefits, these granules could be considered as a supplementary option for lactating mothers experiencing low milk supply, particularly during the early postpartum period when establishing exclusive breastfeeding is crucial. However, it is essential to emphasize that galactagogues should be used as an adjunct to, rather than a replacement for, breastfeeding support and counseling. Ensuring that mothers receive proper guidance on breastfeeding techniques and infant feeding cues is critical to optimizing milk production and reducing the perceived need for galactagogues.

### Limitations

This study has several limitations. Firstly, the results may not be generalizable to all mothers experiencing perceived insufficient lactation, as the study population was limited to those with documented insufficient lactation. While the sample size was adequate to detect statistically significant differences, further studies with larger and more diverse populations are needed to enhance generalizability. Additionally, the 12-week study duration may not have been sufficient to capture long-term effects or the extended safety profile of Ayush-SS granules.

Self-reported data on breastfeeding practices and lactation insufficiency may be subject to recall bias or social desirability bias, potentially affecting accuracy. However, given that both groups were blinded, any reporting errors would likely be similar across the intervention and placebo groups. While efforts were made to ensure objective data collection, some degree of subjective interpretation by investigators during follow-up visits cannot be entirely ruled out.

Furthermore, the study did not comprehensively evaluate all potential adverse effects on both mothers and infants, limiting the scope of the safety assessment. Additionally, the findings are based on a specific demographic, which may not represent broader populations with varying cultural, socioeconomic, and healthcare access differences. Future research with larger, more diverse cohorts and extended follow-up periods is necessary to confirm these findings and evaluate the long-term safety and efficacy of Ayush-SS granules.

## Conclusion

The results indicate that Ayush-SS granules have a statistically and clinically significant effect in improving lactation outcomes and infant weight gain in mothers with documented insufficient lactation. Furthermore, the granules demonstrated a favorable safety profile. Given these findings, Ayush-SS granules may be considered a potential intervention to support breast milk production, particularly during the critical first six months postpartum. However, further large-scale, multisite studies are needed to confirm these results and establish standardized guidelines for their clinical use. Additionally, integrating such interventions with comprehensive breastfeeding support programs may optimize outcomes for mothers and infants.

## Data Availability

No datasets were generated or analysed during the current study.
